# Comparison of Postoperative Bleeding between Application of Polyglycolic Acid Sheet and Primary Closure in Tongue Cancer Patients with Partial Glossectomy

**DOI:** 10.3390/dj8030085

**Published:** 2020-08-03

**Authors:** Satoshi Fukuzawa, Kenji Yamagata, Yuuma Hasegawa, Naomi Ishibashi-Kanno, Fumihiko Uchida, Toru Yanagawa, Hiroki Bukawa

**Affiliations:** Department of Oral and Maxillofacial Surgery, Institute of Clinical Medicine, Faculty of Medicine, University of Tsukuba, 1-1-1 Tennodai, Tsukuba, Ibaraki 305-8575, Japan; y-kenji@md.tsukuba.ac.jp (K.Y.); y.h.d1100@gmail.com (Y.H.); greened_amethyst829@hotmail.com (N.I.-K.); uchiyamada1031@yahoo.co.jp (F.U.); ytony@md.tsukuba.ac.jp (T.Y.); bukawah-cuh@umin.ac.jp (H.B.)

**Keywords:** polyglycolic acid sheet, primary closure, oral cancer, bleeding, partial glossectomy

## Abstract

The technique of covering a mucosal defect with fibrin glue and a polyglycolic acid sheet (MCFP) for the resection of mucosa is applied in oral cancers. The MCFP technique for partial glossectomy provides faster relief from postoperative pain and the prevention of scar contracture, unlike primary closure. However, it has a major complication of postoperative bleeding. This study sought to compare postoperative bleeding between the MCFP technique and primary closure. We designed a retrospective study with a cohort of 57 patients who underwent partial glossectomy with the MCFP technique or primary closure. Our primary predictor variable was the wound closure procedure (primary closure or the MCFP technique). The primary outcome variable was postoperative bleeding, and the other variables were patient characteristics, excision area and depth, tooth contact for the wound, and antithrombotic therapy. Statistical evaluation was performed with Pearson’s chi-squared test, Welch’s t-test, and multiple logistic regression. *P* < 0.05 was considered statistically significant. The MCFP technique was selected for cases with a large excision area (1433 vs. 963 mm^2^, *P* = 0.029). Total postoperative bleeding occurred in 10 of 57 patients (MCFP technique: 7 of 37 cases; primary closure: 3 of 20 cases). There was no significant difference in bleeding between the two groups (*P* = 0.71). Postoperative bleeding was significant in patients with antithrombotic therapy (MCFP: 40% vs. primary closure: 2%, *P* = 0.0024). Postoperative bleeding timing was significantly different in the MCFP technique (6.4 days) from that of primary closure (1 day; *P* = 0.0076). Postoperative bleeding was not associated with the MCFP technique or primary closure. However, postoperative bleeding with the MCFP technique occurred later than that with primary closure. The MCFP technique is not recommended for patients on antithrombotic therapy.

## 1. Introduction

In recent years, polyglycolic acid (PGA) sheets have been used to close pulmonary fistulas during lung surgery, to prevent pancreatic fistulas after pancreatectomy, to prevent leakage of lymph from a fistula, and to prevent the leakage of bile [1,2,3]. Its application in head and neck surgery was reported by Asato et al., who called this technique mucosal defect covered with fibrin glue and PGA sheet (MCFP) [4]. Partial glossectomy is a common surgical procedure in the treatment modalities of T1 or T2 tongue carcinoma classified by the UICC 8th TNM classification. Takeuchi et al. reported that the MCFP technique for partial glossectomy was advantageous in terms of the rapid relief of postoperative pain and the prevention of scar contracture compared to primary closure [5]. Hence, the MCFP technique is a key treatment option for partial glossectomy. However, there have been reports of postoperative bleeding after partial glossectomy. Among them, postoperative bleeding after partial glossectomy is a serious complication and is one of the major risk factors. Here, we report a comparative study of postoperative bleeding between the MCFP technique and primary closure.

## 2. Materials and Methods

### 2.1. Study Approval

This study was conducted in accordance with the Declaration of Helsinki and was approved by the Institutional Review Board of Tsukuba Hospital. Informed consent was waived due to the retrospective nature of the study (No. H29-258, 7 August 2018).

### 2.2. Study Design and Sample

We retrospectively evaluated consecutively diagnosed patients with oral squamous cell carcinoma who underwent partial glossectomy between 2012 and 2019 at the Department of Oral and Maxillofacial Surgery, University of Tsukuba Hospital (Ibaraki, Japan). We evaluated the clinical records of these patients. 

Excisions were performed with a safety margin of 10–15 mm horizontally and vertically from the tumor. Blood vessels were ligated and cauterized with an electric scalpel to confirm hemostasis.

At the end of the procedure, preoperative and postoperative blood pressure values were the same, confirming that the bleeding was arrested. Some patients underwent an additional neck dissection.

### 2.3. Primary Closure Group

The ends of the wounds were sutured, and, for cases that could be closed tension free, primary closure was selected. The primary closure group included those whose wounds were sutured with 4–0 soft nylon threads. From the second day after surgery, oral intake of soft food was permitted.

### 2.4. MCFP Group

**Figure 1:**
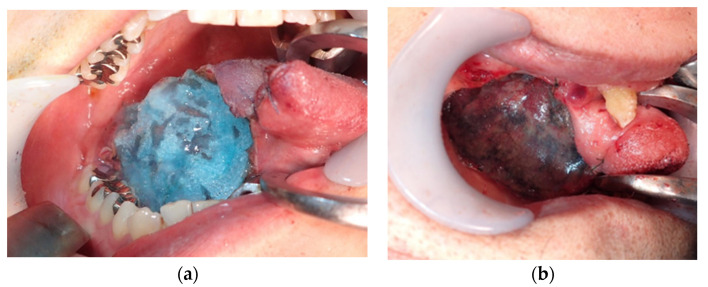
Polyglycolic acid (PGA) sheet attached to the wound (**a**); the fibrin glue was sprayed onto the PGA sheet (**b**).

The MCFP group was defined by those whose wounds were not closed by primary closure alone. The procedure of the MCFP group was as follows: the PGA sheets (Neoveil, Gunze Ltd., Osaka, Japan) and fibrin glue (Beriplast P Combi-Set Tissue adhesion, CSL, PA, USA) were affixed to the open wound not closed by primary closure alone. A PGA sheet with a thickness of 0.15 mm was used. After hemostasis of the wound, a small amount of fibrinogen solution of the fibrin glue was rubbed onto it using a 1 mL syringe. Then, a PGA sheet cut slightly smaller than the wound was affixed, and a mixture of 3 mL each of fibrinogen and thrombin of the fibrin glue was sprayed onto the sheet and the surrounding area using a specialized spray kit. The hardened, excess fibrin glue was removed with scissors (Figure 1). Feeding was by nasogastric tube, and non-liquid oral intake started on postoperative day 7. The postoperative observation period after discharge was once a month in the first year.

### 2.5. Study Variables

The primary predictor variable in this study was the wound procedure (primary closure or the MCFP technique). The patients were divided into binary subgroups of the primary closure group or the MCFP group. The primary outcome variable was postoperative bleeding, and other variables were patient characteristics, excision area and depth, tooth contact for the wound, and antithrombotic therapy. The excision area was calculated using the following formula: length × width × π/4 [5]. The length, width, and depth of the extracted and formalin-fixed specimens were measured. “Tooth contact” was defined as a situation where the lower jaw had contact with the wound. In the case of strong contact, the edges of the teeth were polished. Some patients also received antithrombotic therapy. Cases that needed heparinization, such as warfarin, were heparinized. The heparin was finished around 8 h pre-operation and started after confirming hemostasis on the next day. In cases where heparinization was not required, antithrombotic drugs were discontinued 24 h before surgery. After the operation, if hemostasis was achieved, antithrombotic therapy was restarted on the next day after the surgery.

### 2.6. Data Analysis

Background factors were divided into two groups: the MCFP technique group and the primary closure group. Continuous variables were examined using Pearson’s chi-square test. The subjects were divided into two groups: patients who experienced postoperative bleeding (bleeding group) and patients who did not experience postoperative bleeding (no bleeding group). Patients’ characteristics were compared between groups. 

Multivariate logistic regression analysis was performed on these factors and the number of days of postoperative bleeding was compared between the two groups using Welch’s t-test. All statistical analysis was performed using JMP Pro14 (SAS Institute Inc., Cary, NC, USA). A *P* value < 0.05 was considered statistically significant.

Patients who underwent any medical hemostatic procedure for postoperative bleeding were categorized as “bleeding group.” Patients who did not require procedure for postoperative bleeding were categorized as “no bleeding group.” In the case of postoperative bleeding that was difficult to resolve using primary hemostasis alone, local dental anesthetic containing 1/100000 units of epinephrine, which has vasoconstrictive effects, was infiltrated at doses of 1.8–3.6 mL around the wound.

## 3. Results

### 3.1. Background Factors

The 57 cases enrolled in this study comprised 29 males and 28 females, aged 29 to 93 years (average age: 63.9 years). The MCFP group had 37 cases and the primary closure group had 20 cases. The mean excision area was 1433 mm^2^ in the MCFP group and 963 mm^2^ in the primary closure group. There was a significant difference in excision area between the MCFP group and the primary closure group (*P* = 0.029) (Table 1). The mean depth of excision was 14 mm in the MCFP group and 11 mm in the primary closure group. As a local factor, tooth contact cases were 33 (89%) in the MCFP group and 17 (85%) in the primary closure group. Antithrombotics were administered in four cases (11%) in the MCFP group and one case (5%) in the primary closure group. There were three cases which were converted to preoperative heparin intravenously. Of these five cases (of both groups), three cases received preoperative heparin intravenously, which lasted 8 h preoperatively. Antithrombotic drugs were stopped on the day of the surgery for the two other cases.

Nevertheless, if this was not sufficient to control bleeding, cauterizing with an electric scalpel, administering intravenous tranexamic acid, or additional suturing of the wound was performed as required.

### 3.2. Postoperative Bleeding

Postoperative bleeding occurred in seven (19%) of the 37 cases in the MCFP technique and in three (15%) of the 20 cases of primary closure (*P* = 0.71) (Table 2). All cases of postoperative bleeding occurred in males. This was considered to be scientifically less directly associated with postoperative bleeding; therefore, gender was excluded from the statistical analysis. On the other hand, postoperative bleeding occurred in four (80%) of the five antithrombotic cases. There was a significant difference in postoperative bleeding between antithrombotic cases and non-antithrombotic cases (*P* = 0.0024). In these antithrombotic cases, three (75%) of the four cases had postoperative bleeding in the MCFP technique. Tooth contact cases were nine (90%) in the bleeding group and 41 (87%) in the no bleeding group (Table 2).

The mean number of days of postoperative bleeding was 6.4 days in the MCFP group and one day in the primary closure group. Welch’s *t*-test showed that the number of days of postoperative bleeding in the MCFP group was significantly higher than that in the primary closure group (*P* = 0.0076). Two of the 10 bleeding cases needed emergency surgery under general anesthesia to arrest hemorrhage. One of these patients was from the MCFP technique group, and the other was from the primary closure group.

### 3.3. Multivariate Regression Analysis of Postoperative Bleeding

We performed a logistic regression analysis of the age, MCFP group, excision area, depth, tooth contact, and antithrombotic treatment. The antithrombotic treatment was significantly associated with postoperative bleeding (*P* = 0.011). The other variables were not significant (Table 3).

## 4. Discussion

Primary closure has been performed for small mucosal defects in partial glossectomy. Various methods are used to make up for tissue deficits in cases in which primary closure is comparatively difficult to perform but not large enough to require free flap reconstruction. For example, partial mucous membrane transplantation, skin grafting of the intermediate layer, and covering with various artificial materials like an artificial dermis have been performed. An artificial dermis has been widely used for covering mucosal defects in oral and maxillofacial surgical procedures. However, the disadvantage of an artificial dermis is that it has poor adhesiveness and extensibility and requires tie-over compression [6]. Free skin graft procedures cause significant donor site morbidity. PGA sheet is a bioabsorbable suture reinforcement consisting of polyglycolide, made of nonwoven fabric with some elasticity. The MCFP technique has been reported to be useful in preventing postoperative bleeding, reducing pain, and suppressing scar contraction [7]. On the other hand, some reports showed postoperative bleeding in the MCFP technique [8,9,10,11]. In the present study, there was no significant difference in the occurrence of postoperative bleeding between primary closure and the MCFP technique. However, there was a significant difference in bleeding timing between the two groups (*P* = 0.0076). The bleeding occurred on postoperative day 1 in the primary closure group and after 6.4 days in the MCFP group. Results of previous studies on the MCFP technique are similar to our results. The mean postoperative bleeding time after partial glossectomy by the MCFP technique was 7.5 (1–18) days and the bleeding rate was 24% [8,9,10,11] (Table 4). Although attention is required for bleeding on postoperative day 1 in the primary closure technique, it is required for up to 1 week postoperatively in the MCFP technique. 

Adhesive force occurs approximately 1 min after fibrinogen and thrombin are mixed, increases after approximately 12 h, and becomes firm in 36 to 48 h [12]. However, postoperative bleeding has occurred in the MCFP technique after more than 48 h. Taniguchi et al. reported that the postoperative bleeding was caused by the irritation of the wound by tooth contact [8]. In the case of tongue cancer, Suzuki et al. reported that the PGA sheets often dropped off early and exposed the wound with insufficient epithelialization, thus causing bleeding from tooth contact in speech, eating, and swallowing [9]. Moreover, Taniguchi et al. reported that three of six cases of postoperative bleeding occurred before oral ingestion and three after oral ingestion [8]. In our study, there was no significant difference in bleeding from tooth contact. Although the early drop-off of the PGA sheet was not observed in our study due to nutrition by nasogastric tube for one week, bleeding may occur from drop-off after one week at the time of beginning oral intake.

In the present study, postoperative bleeding occurred in four (80%) of five antithrombotic cases and three (75%) of four bleeding cases of the MCFP group. Therefore, the MCFP technique is not recommended for partial tongue cancer resection in patients on antithrombotic therapy. For patients with antithrombotic therapy, it may be appropriate to use primary closure that can be sewn, and if the wound is too large to be appropriate for primary closure, conventional skin grafting or raw surface with tie-over may be adopted. In this study, bleeding was not associated with the area and depth of resection. This is probably because when the excision area was larger or the depth was greater than present selected cases, reconstructive surgery with free flaps is applied.

Yoshifuku et al. reported that postoperative bleeding in palatine tumors in the MCFP technique occurred in two of three cases, which required hemostasis under general anesthesia. The bleeding times for the two cases were 12 and 19 days, respectively [10]. In our study, one patient from the MCFP technique group needed emergency surgery under general anesthesia to arrest hemorrhage. It is recommended to envisage the possibility of emergency surgery for hemostasis before tongue cancer surgery. When applying the MCFP technique, sufficient measures must be taken to control bleeding until the wound surface is completely covered with epithelium. It therefore seems that it is appropriate to manage these patients by hospitalization for about 10 days after MCFP surgery. In addition, Okuyama et al. reported the risk of developing granuloma-like neoplasm with the application of the MCFP technique of oral mucosa [13]. We did not experience such a case, but we must also take care to avoid granuloma-like neoplasm.

This study was a single center study with a limited number of cases. We would like to increase the number of cases and consider research at other facilities in the future.

## 5. Conclusions

Postoperative bleeding was not associated with the MCFP technique or with primary closure. However, postoperative bleeding in the MCFP technique occurred later than in primary closure. The MCFP technique is not recommended for patients on antithrombotic therapy. 

## Figures and Tables

**Table 1:** Background factors.

Variable	MCFP Group (*n* = 37)	Primary Closure Group (*n* = 20)	*P*-Value
Gender			
Male (cases)	18	11	
Female (cases)	19	9	0.78
Age			
Mean ± SD (years)	63.3 ± 14.1	65.1 ± 10.0	0.68
T classification			
T1	18	14	
T2	19	6	0.16
Excesion factor			
Area, mean ± SD (mm^2^)	1433 ± 647	963 ± 572	0.029 *
Depth, mean ± SD (mm)	14.0 ± 5.1	11.1 ± 2.8	0.065
Local factor			
Tooth contact cases (%)	33 (89)	17 (85)	0.69
General factor			
Antithrombotic therapy cases (%)	4 (11)	1 (5)	0.65
Postoperative bleeding (%)	7 (19)	3 (15)	0.71

MCFP, Mucosal defect covered with fibrin glue and polyglycolic acid sheet; SD, standard deviation. * *P* < 0.05.

**Table 2:** Postoperative bleeding.

Variable	Bleeding Group (*n* = 10)	No Bleeding Group (*n* = 47)	*P*-Value
Age, mean ± SD (years)	68.3 ± 7.6	63.0 ± 13.3	0.33
MCFP technique (cases)	7	30	0.71
Area, mean ± SD (mm^2^)	1152 ± 570	1293 ± 650	0.6
Depth, mean ± SD (mm)	14.6 ± 5.1	12.6 ± 4.3	0.31
Tooth contact cases (%)	90	87	0.81
Antithrombotic therapy cases (%)	4 (40)	1 (2.1)	0.0024 *

MCFP: Mucosal defect covered with fibrin glue and polyglycolic acid sheet; SD: standard deviation; * *P* < 0.05.

**Table 3:** Multivariate logistics regression about postoperative bleeding.

	B	Wald	*P*-Value	95% CI
Age	0.0076	0.067	0.8	−0.050–0.065
MCFP technique	−0.19	0.16	0.69	−1.1–0.72
Average area	−0.00022	0.12	0.72	−0.0014–0.0010
Average depth	0.12	1.7	0.19	−0.063–0.31
Tooth contact	0.78	0.81	0.37	−0.92–2.5
Antithrombotic therapy	1.86	6.4	0.011 *	0.42–3.3

95% CI: confidence interval. * *P* < 0.05.

**Table 4:** Literature review of bleeding in partial glossectomy by the MCFP technique.

Author	Year	N	Bleeding Cases (%)	Mean Number of Days of Bleeding
				(Duration)
Taniguchi et al. [8]	2012	15	6 (40)	9.3 (5–18)
Suzuki et al. [9]	2015	26	9 (34.6)	7.2 (5–10)
Yoshifuku et al. [10]	2016	12	1 (8.3)	1
Hirobe et al. [11]	2017	22	4 (18.2)	9 (5–13)
Our study	2020	37	7 (18.9)	6.4 (1–12)
Total		112	27 (24.1)	7.5 (1–18)
